# A135 INCREASED USE OF PSYCHIATRIC MEDICATION USE FOLLOWING DEVELOPMENT OF CELIAC DISEASE AUTOIMMUNITY

**DOI:** 10.1093/jcag/gwae059.135

**Published:** 2025-02-10

**Authors:** Q Goddard, J A King, S Coward, T Williamson, G G Kaplan

**Affiliations:** Community Health Sciences, University of Calgary, Calgary, AB, Canada; Community Health Sciences, University of Calgary, Calgary, AB, Canada; Community Health Sciences, University of Calgary, Calgary, AB, Canada; Community Health Sciences, University of Calgary, Calgary, AB, Canada; Community Health Sciences, University of Calgary, Calgary, AB, Canada

## Abstract

**Background:**

Celiac disease (CeD) autoimmunity is characterized by new positivity for tissue transglutaminase antibodies (TTG) with or without pathologic confirmation. CeD autoimmunity is associated with an increased risk of psychiatric disorders. However, patterns in treatment for psychiatric conditions in relation to diagnosis of celiac autoimmunity remain unclear.

**Aims:**

To determine trends in psychiatric medication use in individuals with CeD autoimmunity before and after TTG positivity.

**Methods:**

A population-based cohort of individuals with incident CeD autoimmunity (*n* = 14,324; 9,180 females) between April 2015 and March 2023 in Alberta was linked to medication dispensation records. Individuals were classified as children (≤ 12), young adults (13–24), adults (25-64), or seniors (≥ 65) according to age at time of TTG positivity. Medications were categorized by anatomical therapeutic chemical codes: N06A (antidepressants), N05B (anxiolytics), and N05A (antipsychotics). Dispensation data was collected for up to five years before and after TTG positivity. The proportion of days covered (PDC) was calculated as the number of days a drug was prescribed over the follow-up period, per patient. McNemar’s test (for proportions) and Wilcoxon signed rank tests (for PDC) were used to compare medication use before vs after TTG positivity.

**Results:**

Antidepressants were prescribed to 36% of the cohort, anxiolytics to 21%, and antipsychotics to 8% at least once during the study period. A significantly greater proportion of the cohort dispensed antidepressants (20% vs 26%) and antipsychotics (4% vs 6%, both *p* < 0.001) after TTG positivity, while a significantly lower proportion dispensed anxiolytics (12% vs 11%, *p* = 0.01) after TTG positivity. A greater proportion of all age categories and sexes dispensed antidepressants after TTG positivity, except for seniors (*p* = 0.84). The largest increase occurred in young adults, with 24% vs 36% dispensing antidepressants pre- vs post-TTG positivity. PDC significantly increased post-diagnosis for antidepressants (11% vs 35%), anxiolytics (2% vs 4%), and antipsychotics (6% vs 23%, all *p* < 0.001). For all drug classes, all age groups and sexes had a significantly higher PDC post-TTG, except for children (*p* = 0.20) and seniors (*p* = 0.38) with anxiolytics.

**Conclusions:**

Psychiatric medication use is common in individuals with CeD autoimmunity, with increased use following TTG positivity. While the proportion of individuals on medication is only marginally greater following TTG positivity, we observe increased reliance (i.e., PDC) on medication following TTG positivity. These findings suggest enhanced monitoring for psychiatric comorbidities is warranted in this population.

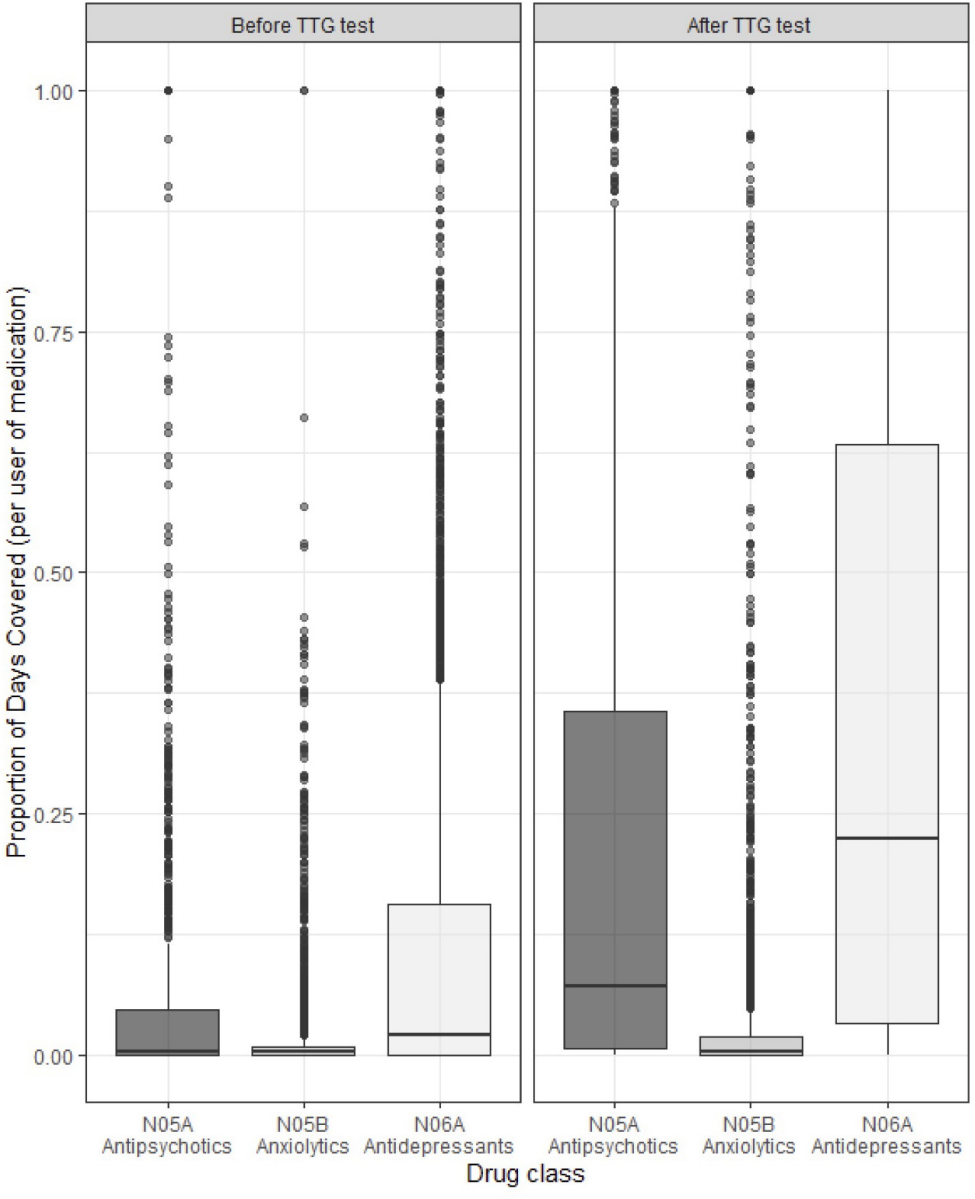

Individual-level PDC by medication class, five years before and after TTG test.

**Funding Agencies:**

CIHR

